# A multidisciplinary and structured approach for comprehensive evaluation of functional outcomes, adverse events, psychosocial outcomes and health-related quality of life after local therapy for bone sarcoma in children: protocol for a cross-sectional study

**DOI:** 10.3389/fped.2025.1534153

**Published:** 2025-04-15

**Authors:** Leonie G. Tigelaar, Lianne M. Haveman, Willem P. Bekkering, Irene L. B. Oude Lansink, Christel D. Rohrich, Hinke Van der Hoek, Laura R. Beek, Jennifer Van Dijk, Marjolein E. M. Langemeijer, Relinde W. Slooff-Lentink, Alied M. Van der Aa-Van Delden, Heleen Maurice-Stam, Annemarie M. L. Peek, Helena J. H. Van der Pal, Maria M. W. Koopman, Leontien C. M. Kremer, Stijn J. Westerbos, Harm Van Tinteren, Jos A. M. Bramer, Michiel A. J. Van de Sande, Martha A. Grootenhuis, Hendrik W. B. Schreuder, Johannes H. M. Merks

**Affiliations:** ^1^ Princess Máxima Center for Pediatric Oncology, Utrecht, Netherlands; ^2^ Department of Pediatric Rehabilitation, Wilhelmina Children’s Hospital, University Medical Center Utrecht, Utrecht, Netherlands; ^3^ Department of Orthopedic Surgery and Sports Medicine, Amsterdam Movement Sciences, Cancer Center Amsterdam, Amsterdam University Medical Centers, University of Amsterdam, Amsterdam, Netherlands; ^4^ Department of Orthopedic Surgery, Leiden University Medical Center, Leiden, Netherlands; ^5^ Department of Orthopedic Surgery, Radboud University Medical Center, Nijmegen, Netherlands; ^6^ Division of Imaging and Oncology, University Medical Center Utrecht, Utrecht University, Utrecht, Netherlands

**Keywords:** adverse events, bone sarcoma, functional outcome, orthopedic surgery, pediatric oncology, study protocol, quality of life

## Abstract

**Introduction:**

Bone sarcoma patients face intensive treatment, including life-changing local therapy, which impacts both short- and long-term functioning. Moreover, bone sarcoma survivors experience the highest burden of adverse events of all childhood cancer survivors. To address these issues, we set up a structured multidisciplinary outpatient follow-up clinic for patients who completed treatment and integrated this clinic into the standard of care. This study protocol describes the methodology of a cross-sectional study that aims to systematically report the functional outcomes, adverse events, psychosocial outcomes and health-related quality of life of the cohort seen at this clinic.

**Methods and analysis:**

Participants are recruited at the multidisciplinary follow-up clinic and their consent is obtained. Standard of care clinical assessments serve as the primary data source for this study. Furthermore, additional research assessments are performed to further expand our knowledge. Assessments are structured by standardized assessment sets that we developed based on literature review and joint national expertise in bone sarcoma care. The sets comply with international guidelines such as the World Health Organization's International Classification of Functioning, disability and health, and include a combination of patient-reported, clinician-reported and performance-based outcome measures for comprehensive representation of outcomes.

**Discussion:**

This study will generate valuable knowledge on the functional outcomes, adverse events, psychosocial outcomes and quality of life of a national cohort of pediatric bone sarcoma patients in follow-up care. By aligning additional research assessments with standardized patient care, a comprehensive range of outcomes will be obtained while minimizing the patient's burden. Moreover, this protocol may serve as a template for clinics and research internationally, allowing for the merging of standardized outcome data in such rare disease. This will facilitate the optimization of current patient care and inform the important shared decision-making process for local treatment in future patients.

## 1 Introduction

Osteosarcoma and Ewing sarcoma are the most common malignant bone tumors in children with approximately 35 new diagnoses per year in the Netherlands (1). Treatment consists of neoadjuvant chemotherapy, local therapy and adjuvant chemotherapy. Local therapy is primarily surgical, though Ewing sarcoma patients may also receive radiotherapy based on tumor characteristics or as a standalone treatment in specific cases. Surgical options consist of limb-sparing surgery, including tumor resection followed by reconstruction using allografts, autografts, and/or prostheses, and ablative surgery (i.e., amputation or rotationplasty). The choice of surgery depends on oncological considerations, such as involvement of surrounding tissues and presence of metastases, as well as patient preferences. Given the complexity of treatment, a multidisciplinary approach is crucial for optimal care. Oncologists, orthopedic surgeons, pathologists, radiologists, rehabilitation physicians, physical therapists, and psychologists all play critical roles and must collaborate closely to maximize treatment and rehabilitation outcomes.

Despite this, the intensive treatment regimen of bone sarcoma patients causes them to experience the highest burden of adverse events among pediatric cancer survivors (2). This is largely due to the life-changing local therapy, which impacts both short- and long-term outcomes across multiple domains, i.e., functional outcomes, psychosocial outcomes, and/or overall health-related quality of life (HR-QOL) (3). Consequently, follow-up care for osteosarcoma and Ewing sarcoma patients is multifaceted and complex. In the recent past, our national comprehensive childhood cancer center, and the individual academic centers before the centralization of care, lacked a cohesive multidisciplinary approach to follow-up. This resulted in fragmented care. Typically, patients would visit a pediatric oncologist or late effects specialist regularly and, depending on the type of surgery, occasionally visit an orthopedic surgeon. Additional healthcare professionals, such as a physical therapist, rehabilitation physician and/or psychologist, were called upon indication.

To enhance the quality of follow-up care, integrate multidisciplinary expertise in our center, and streamline policy coordination, we set up a multidisciplinary outpatient follow-up clinic for bone sarcoma patients after completion of antitumor treatment. Through extensive research of literature supplemented with the available national expertise in bone sarcoma care, we identified the domains requiring evaluation during the clinic. As a result, the evaluated outcomes include important aspects of functional outcomes, adverse events, body image issues, emotional distress, social functioning, and overall HR-QOL. To assess these outcomes, we use a combination of patient-reported outcome measures (PROMs), clinician-reported outcome measures (ClinROMs) and performance-based outcome measures (PerBOMs). Standard of care investigations that are part of routine follow-up, as prescribed by international collaborative studies on osteosarcoma and Ewing sarcoma, are integrated according to the respective (inter)national bone sarcoma and late effects guidelines (4–6).

Following the domains requiring evaluation, the clinic includes carousel consultations with a pediatric oncologist or late effects specialist, orthopedic surgeon, rehabilitation physician, physical therapist, and psychologist. Since most follow-up patients were anticipated to benefit from screening by a physical therapist for mobility, strength, and functional movement—while not universally requiring the specialized adjustments provided by occupational therapists—physical therapists were included in the standard team. Evaluation of arm-hand function, typically performed by an occupational therapist, was incorporated into the physical therapist's role when necessary. Similarly, radiation oncologists were not included in the standard team, as the majority of patients were estimated to have not received radiation. Instead, radiotherapy-related late effects were monitored by the pediatric oncologist and orthopedic surgeon. In cases of specific questions or concerns, occupational therapists, radiation oncologists, or other relevant professionals were consulted or referred to as needed. Additionally, given the overlap in physical assessment, physical therapists and rehabilitation physicians conducted joint consultations.

Each multidisciplinary clinic is preceded by a preclinic team meeting among the healthcare professionals in the morning and followed by a postclinic team meeting in the late afternoon, all occurring on the same day. These meetings guarantee dedicated moments for the exchange of findings and interdisciplinary consultation and coordination. This, in turn, promotes effective collaboration and optimal coordinated patient care including streamlined referrals when indicated.

The implementation of this clinic as part of our standard of care, with its comprehensive and structured information gathering in a multidisciplinary format, presents us with an opportunity to systematically acquire valuable insights into the outcomes of a national cohort of bone sarcoma patients during follow-up. Therefore, in this study we aim to systematically evaluate functional outcomes, adverse events, psychosocial outcomes, and HR-QOL in patients treated for pediatric bone sarcoma and seen at the multidisciplinary follow-up clinic. Additionally, we will evaluate the added value of the multidisciplinary and structured care.

The findings of this study will be used to optimize follow-up care for bone sarcoma patients and survivors by identifying common challenges after treatment and refining the multidisciplinary approach to better address their needs. In addition, these insights will enhance the shared decision-making process among newly diagnosed patients, parents, and healthcare professionals by providing more comprehensive outcome data to guide discussions on local therapy options and long-term expectations.

## 2 Methods and analysis

### 2.1 Study design

This is a cross-sectional nationwide study conducted at the Princess Máxima Center for pediatric oncology. Since June 2018, all childhood cancer care in the Netherlands has been centralized at the Princess Máxima Center, the national comprehensive childhood cancer center. Prior to this centralization, pediatric oncology care was provided at one of six university medical centers across the country. The Princess Maxima Center treats all children and adolescents (<18 years) with cancer in the Netherlands. Additionally, the center provides lifelong follow-up and late effects care, ensuring continuity of specialized care after childhood cancer well into adulthood.

Prior to full integration into standard care, the multidisciplinary follow-up clinic was tested with two patients to assess the feasibility of assessments and logistics. Since no challenges were encountered, the clinic was subsequently incorporated into standard follow-up care. From that point forward, all patients attending the multidisciplinary follow-up clinic have been screened for eligibility to participate in this study. Recruitment started November 18th, 2021, and the cohort is expected to be concluded within four years.

### 2.2 Participants

For inclusion a subject must meet all of the following criteria: (1) patient/survivor of osteosarcoma or Ewing(-like) sarcoma at least two years after diagnosis, (2) under 18 years of age at diagnosis, (3) date of diagnosis from 2003, (4) completed antitumor treatment without evidence of disease, (5) had a tumor located in the upper extremity (including the shoulder girdle), lower extremity or pelvis (including the sacrum), and (6) received local therapy with surgery and/or radiotherapy. Patients who do not meet all criteria, patients with a relapse or on palliative treatment, and patients with medical conditions unrelated to the local therapy of the primary tumor that severely limit physical activities are excluded from the study.

### 2.3 Sample size

Based on the national incidence of osteosarcoma and Ewing sarcoma in the pelvis and extremities in patients under 18 years (approximately 25 patients per year) and a survival rate of around 65%, we estimated that 16 new patients per year would reach follow-up without evidence of disease two years after diagnosis (calculation supported by data from the Dutch Childhood Oncology Group national Childhood Cancer Registry and Long-Term Effects After Childhood Cancer Registry). Given the expected inclusion period, covering patients diagnosed between 2003 and mid-2023, we estimated a total of 330 eligible patients in the Netherlands. At the time of centralization of childhood oncology care to the Princess Máxima Center in 2018, 150 patients were not transferred from the UMCs to the Princess Máxima Center for follow-up and late effects care (out of approximately 280 in total). This was primarily because two of the four national orthopedic oncology UMCs retained follow-up care for their cohorts. As a result, we anticipated inviting approximately 180 patients to the clinic. Considering a non-participation rate of 20%, we estimated that around 145 patients would be included in the study: 20 with tumors in the upper extremity, 20 with pelvic tumors, and 105 with lower extremity tumors.

### 2.4 Informed consent

**Figure 1:**
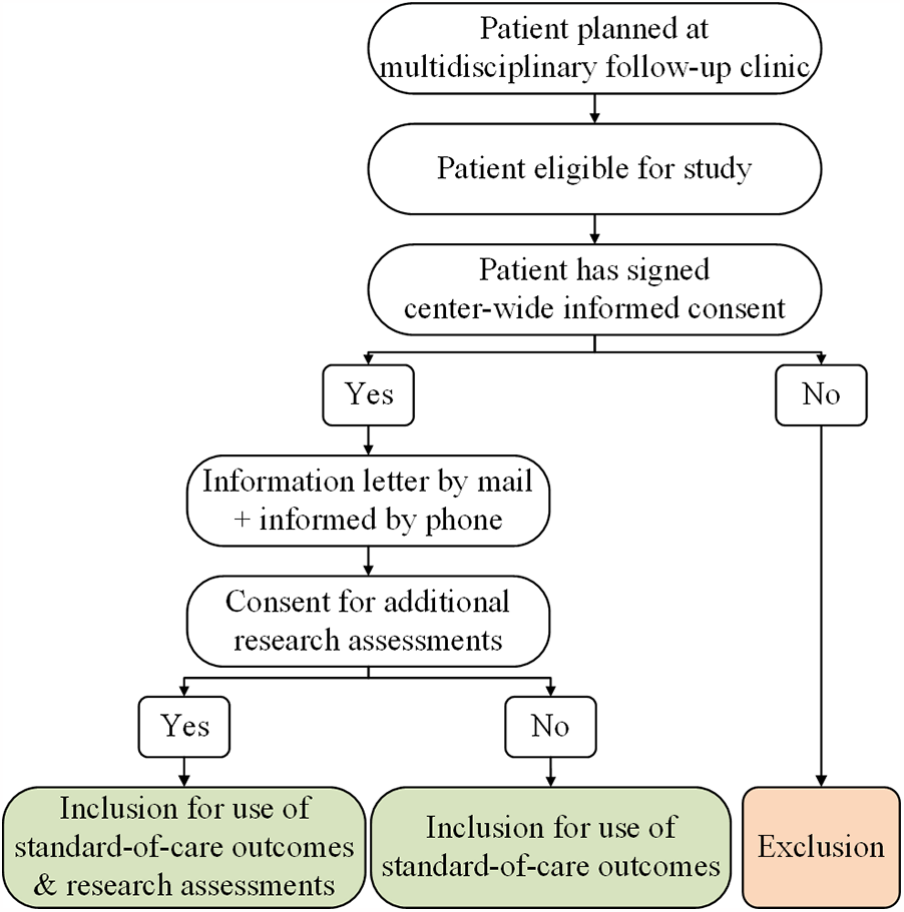
Informed consent process.

**Figure 2:**
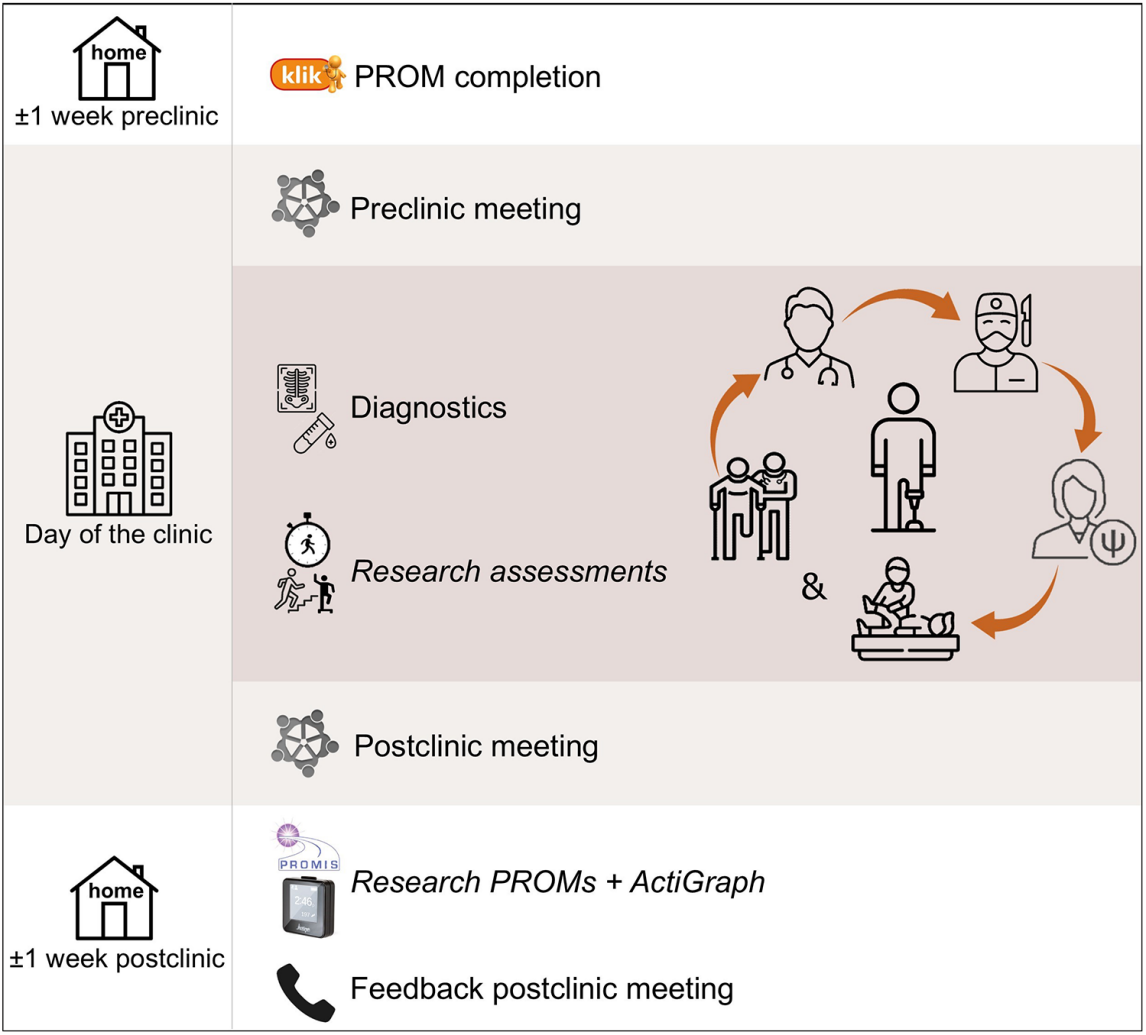
Setup of multidisciplinary clinic including incorporation of research assessments in *italics.*

Our hospital has implemented a center-wide informed consent policy that covers the usage of all clinical data collected throughout a patient's journey. In accordance with this written consent, outcomes derived from the standard care provided at our multidisciplinary follow-up clinic can be used for the study if the patient is considered eligible. In addition, eligible patients are presented with the opportunity to participate in a small set of additional research assessments. Since these assessments go beyond the scope of the clinic's standard care, separate consent is required. Hence, patients are requested to provide written informed consent specifically for the additional research assessments conducted as part of this study (Figure 1). The incorporation of additional research assessments within the setup of the multidisciplinary follow-up clinic is illustrated in Figure 2.

### 2.5 Outcomes

The primary outcome of the study measures functional outcomes as classified by the World Health Organization's (WHO) International Classification of Functioning, disability and health (ICF) (see “conceptual frameworks” below) (7). Secondary outcomes include adverse events, psychosocial outcomes and HR-QOL. Furthermore, we will evaluate the multidisciplinary follow-up clinic by assessing: (1) patient satisfaction with the received care and (2) the output of care, defined as the action points initiated as a direct outcome of the clinic.

### 2.6 Conceptual frameworks

**Figure 3:**
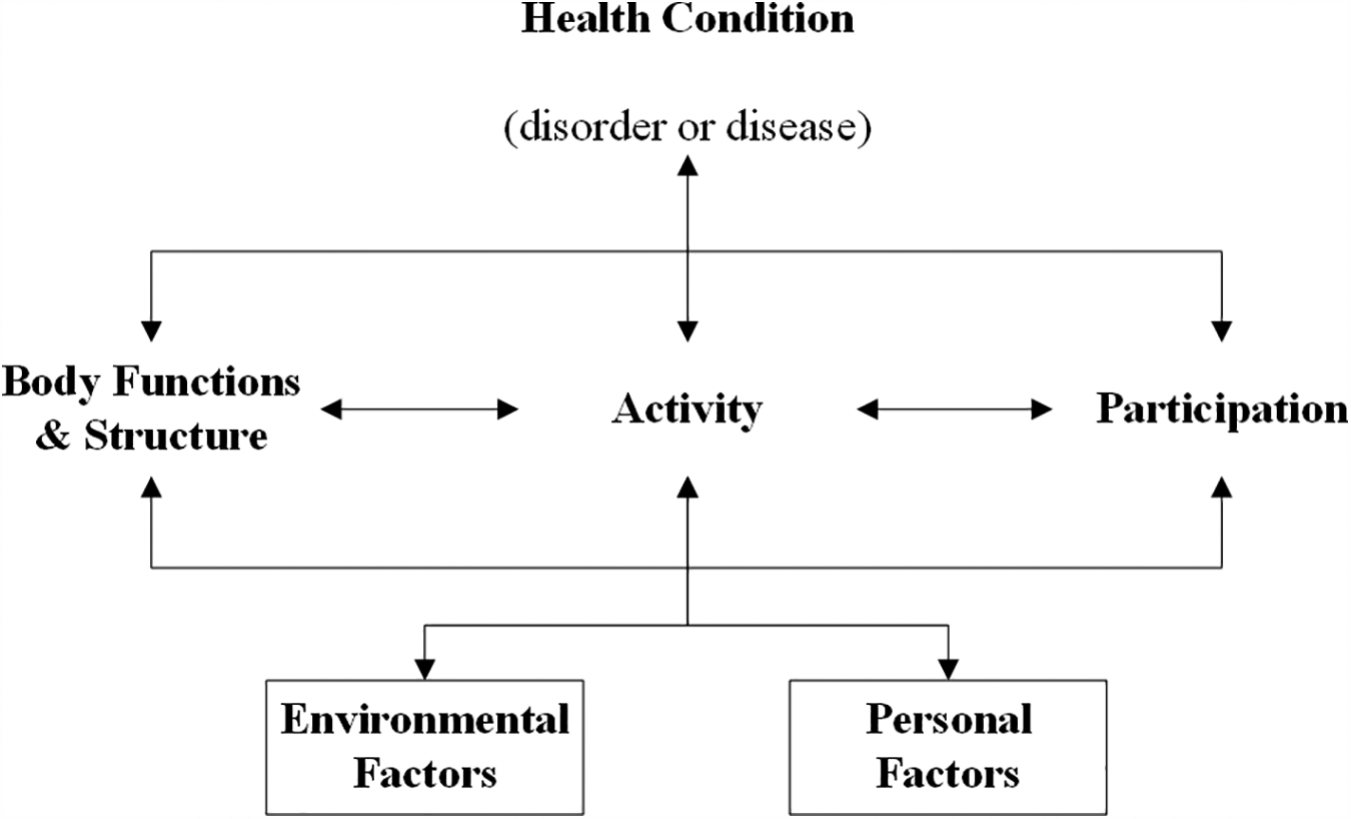
International classification of functioning, disability and health. Original work by “World Health Organization (7). License: CC BY-NC-SA 3.0 IGO”.

Two conceptual frameworks were selected to support the structured evaluation of the outcomes. For the functional outcomes we used the WHO's ICF (7). According to this framework, functioning is classified into three levels. “Body functions and structures” addresses the anatomical parts and physiological functions of body systems. “Activities” refers to the ability to execute tasks, and “Participation” describes to which extend a person can carry out their normal role functioning (Figure 3). Even though this framework was designed to map a patient's overall functioning, including e.g., physical, psychosocial and personal aspects, it is particularly useful in understanding and systematically describing functional outcomes.

Since the ICF describes the various components of psychosocial outcomes and HR-QOL less detailed, we used the framework reported by Anthony et al. to systematically classify and assess these outcomes (8). In this review, items of quality of life measures used in pediatric oncology are analyzed and categorized into major domains, subdomains and identifying concepts, leading to a structured overview of the aspects of HR-QOL in this population.

### 2.7 Measurement of outcomes

Data on study outcomes are derived from: (1) a structured evaluation of standard-of-care assessments at the multidisciplinary follow-up clinic, and (2) additional research assessments.

Assessments already in use in follow-up care or deemed essential but inconsistently assessed or documented, were standardized into a core measurement set to ensure uniformity across patients. Beyond these standard-of-care assessments, we included research-specific evaluations expected to deepen understanding and potentially guide future clinical practice.

#### 2.7.1 Standard of care measurement set

The standard of care assessments conducted at the multidisciplinary follow-up clinic comprise a spectrum of measures that collectively cover the domains important to patients with bone sarcoma. The following aspects were considered in the selection process: suitability for the age of the bone sarcoma population, availability of textual measurements in Dutch (for PROMs), psychometric properties, availability of norm values (if applicable), adequate coverage of relevant domains with minimal overlap, and acceptable patient burden in terms of question content and time investment. Patients are requested to complete the PROMs at home via the KLIK (Quality of Life in Clinical Practice) PROM online portal prior to the clinic visit (9). ClinROMs and PerBOMs are conducted during the clinic itself.

A brief description of the included standard of care assessments per outcome is described below. The detailed properties of the assessments, along with their corresponding domains as described by the ICF (for functional outcomes) and by Anthony et al. (for psychosocial outcomes), are presented in Table 1 (7, 8).

**Table 1:** Properties of the standard of care measurements.

(Sub)domain(s)	Measure	Type	Subscales	Items	Response scale/units	Age (years)	Norms available	Completion time (min)
Functional outcomes^e^								
Body functions & structures	Goniometer	PerBOM	–	Patient dependent^b^	Degrees	All	–	5
	MRC scale	PerBOM	–	Patient dependent^b^	0–5	All	–	5
	Hand-held dynamometer	PerBOM	–	Patient dependent^b^	Newton	≥2	–	5
	Stand on 1 leg	PerBOM	–	–	Seconds	≥2	–	3
	Leg length discrepancy	PerBOM	–	–	Centimetres	All	–	1
	Numeric Rating Scale pain	PROM	–	1	0–10	≥8	No^c^	<1
	PedsQL™ Multidimensional Fatigue Scale	PROM	3	18	5-point Likert	8–30	Yes (10, 11)	3
Activity	Box and block test	PerBOM	–	–	Number in 60 s	≥5	Yes (12–14)	3
	10-meter walk test	PerBOM	–	–	Seconds	≥2	Yes (15, 16)	1
	10-meter run test	PerBOM	–	–	Seconds	≥2	No	1
	TESS—upper extremity	PROM	–	29	5-point Likert + N/A	≥12	–	5
	TESS—lower extremity	PROM	–	30	5-point Likert + N/A	≥12	–	5
Activity & Participation	USER-P	PROM	3	32	6-point Likert 4-point Likert + N/A 5-point Likert	≥10	–	5
Adverse events								
	Henderson classification	ClinROM	–	6	Present/absent	All	–	<1
	CTCAE	ClinROM	7–12^a^	28–53^a^	Grade 1–5	All	–	5
Psychosocial outcomes^f^								
Body image	PedsQL™ Cancer Module: Perceived physical appearance	PROM	–	3	5-point Likert	≥8	–	1
	Body Image Scale	PROM	–	10	4-point Likert	≥16	–	2
Emotional distress	Emotion thermometers	PROM	5	5	0–10	≥8	No^d^	1
Self-esteem	Impact of Cancer—Cancer Survivors	PROM	11	81	5-point Likert	≥18	–	15
Positive psychological function								
Cognitive								
Emotional distress								
Physical function								
Social function								
Relationships								
Health perception								
HR-QOL^f^								
Emotional distress	PedsQL™ Generic Core Scales	PROM	4	23	5-point Likert	5–30	Yes (17–19)	4
Physical function								
Social function								

^a^ Number of subscales and items dependent on the type of local therapy (i.e., surgery, radiotherapy or both) and location of the tumor (i.e., upper extremity, pelvis or lower extremity).

^b^ The assessed joints and muscles depend on the location of the tumor and the local therapy applied.

^c^ The cut-off for tolerable pain threshold was set at a score of four (20).

^d^ Cut-off for further inquiry was set at a score of four (21).

^e^ Stratified by domains according to the World Health Organization's International Classification of Functioning, disability and health (7).

^f^ Stratified by (sub)domains as described by Anthony et al. (8).

ClinROM, clinician-reported outcome measure; CTCAE, common terminology criteria of adverse events; HR-QOL, health-related quality of life; MRC scale, medical research council scale; PedsQL, pediatric quality of life inventory; PerBOM, performance-based outcome measure; PROM, patient-reported outcome measure; TESS, toronto extremity salvage score; USER-P, utrecht scale for evaluation of rehabilitation—participation.

##### 2.7.1.1 Functional outcomes

A standardized physical assessment stratified by affected body part and/or type of surgery, is completed by the physical therapist and rehabilitation physician (see Supplementary Data Sheets S1 and S2). Joints and muscles are structurally examined including range of motion, muscle power, and functional and timed testing.

The box and block test is included for patients with upper extremity tumors to assess lateral movement of the arm in the frontal space, which is crucial in daily life (12, 13). For this test, a patient is asked to transfer as many blocks as possible using one hand from one side of the box, over a 15-centimeter high partition, to the other side of the box within 60 s. The test is performed independently for each hand.

Standing on one leg and the 10-meter walk and 10-meter run tests are performed in patients with pelvic or lower extremity tumors to assess balance, walking and running, which play a crucial role in daily functioning (22).

Patients rate their pain intensity over the past week using a numeric rating scale, where 0 represents no pain and 10 the worst imaginable pain (23).

General fatigue, sleep/rest, and cognitive fatigue are evaluated by the Pediatric Quality of Life Inventory Multidimensional Fatigue Scale (PedsQL™ MFS) self-report (24).

The Toronto Extremity Salvage Score (TESS) assesses the ability to perform daily life activities. This PROM includes separate versions for upper and pelvic/lower extremity tumors (25, 26).

The Utrecht Scale for Evaluation of Rehabilitation—Participation (USER-P) explores the frequency of daily activities, experienced limitations, and patient satisfaction with functioning (27, 28).

##### 2.7.1.2 Collection of adverse events

The Common Terminology Criteria for Adverse Events (CTCAE) is used to report the adverse events resulting from local therapy (surgery and/or radiotherapy) that are encountered at the follow-up clinic (29). This system comprises a large number of events graded on five levels from mild to death. To address the most relevant events in our outpatient clinic population, a selection of CTCAE terms was made stratified by tumor location and type of local therapy (see Supplementary Data Sheet S3). Completion of CTCAE items is distributed based on expertise among the pediatric oncologist/late effects specialist, orthopedic surgeon, and rehabilitation specialist. For example, the orthopedic surgeon assesses musculoskeletal and connective tissue disorders such as decreased joint range of motion, osteonecrosis, and muscle weakness. The rehabilitation specialist evaluates pain, gait abnormalities, and lymphedema, while the pediatric oncologist/late effects specialist assesses gastrointestinal symptoms following radiotherapy.

Failure modes of reconstructions after limb-sparing surgery are recorded by the orthopedic surgeon using the Henderson classification (30). Two versions of this classification are available: one for failure of endoprostheses and another for biological reconstructions.

##### 2.7.1.3 Psychosocial outcomes and HR-QOL

Based on literature review supplemented with psychosocial expertise in our center, the selection of measures for psychosocial outcomes and HR-QOL was focused on coverage of the (sub)domains “emotional distress”, “body image”, “social function” and overall HR-QOL (8).

The Emotion Thermometers tool measures emotional distress in the past week by asking the patient to rate “Distress”, “Depression”, “Anxiety”, and “Anger” on a visual analogue scale, where zero represents none and ten represents extreme (21, 31). A fifth thermometer assesses the “Need for help”, ranging from being able to manage by yourself to being desperately in need of help.

Because of the impact of local therapy on patients' physical appearance, body image is considered crucial in counselling and evaluating patients' well-being. Body image is measured in all patients by a subscale of the Pediatric Quality of Life Inventory Cancer Module (PedsQL™ CM) self-report called “Perceived physical appearance” (24). For patients 16 years and older, this evaluation is supplemented by the Body Image Scale (BIS); a short PROM originally developed for cancer survivors (32).

The Impact Of Cancer–Cancer Survivors (IOC-CS) addresses the impact of childhood cancer on adult life (33). This PROM encompasses a comprehensive assessment of diverse facets of long-term survivorship.

The Pediatric Quality of Life Inventory Generic Core Scales (PedsQL™ GCS) self-report examines overall HR-QOL by including physical functioning, emotional functioning, social functioning and school/work functioning (34). It allows for longitudinal assessment by providing distinct self-report forms for different age groups.

#### 2.7.2 Research measurement set

The measures described below are performed in patients who consent for the additional research measurements of the study. Detailed properties of the assessments are listed in Table 2.

**Table 2:** Properties of the research measurements.

(Sub)domain(s)	Measure	Type	Subscales	Items	Response scale/units	Age (years)	Norms available	Completion time (min)
Functional outcomes^b^								
Body functions & structures	PROMIS Pediatric v2.0 Pain interference	PROM	–	CAT	5-point Likert	8–17	Yes^a^	1
	PROMIS v1.1 Pain interference	PROM	–	CAT	5-point Likert	≥18	Yes^a^	1
	PROMIS Pediatric v2.0 Fatigue	PROM	–	CAT	5-point Likert	8–17	Yes^a^	1
	PROMIS v1.0 Fatigue	PROM	–	CAT	5-point Likert	≥18	Yes^a^	1
Activity	Rate of Perceived Exertion	PerBOM	–	–	0–10	≥5	–	<1
	6-Minute Walk Test	PerBOM	–	–	Meters	≥5	Yes (35, 36)	7
	ActiGraph GT9X Link	PerBOM	–	–	Number of steps Activity intensity	≥5	Yes	7 days
	Lateral Step Up Test	PerBOM	–	–	Number in 15 s	≥5	No	1
	PROMIS Pediatric v2.0 Physical function—Mobility	PROM	–	CAT	5-point Likert	8–17	Yes	1
	PROMIS Pediatric v2.0 Physical function—Upper extremity	PROM	–	CAT	5-point Likert	8–17	Yes	1
	PROMIS v1.2 Physical function	PROM	–	CAT	5-point Likert	≥18	Yes	1
	Timed Up and Down Stairs	PerBOM	–	–	Seconds	≥5	No	1
Psychosocial outcomes^c^								
Cognitive	PROMIS Pediatric v1.0 Cognitive function	PROM	–	43	5-point Likert	8–17	Yes^a^	7
	PROMIS v2.0 Cognitive function 8a	PROM	–	8	5-point Likert	≥18	Yes^a^	1
Emotional distress	PROMIS Pediatric v.2.0 Anxiety	PROM	–	CAT	5-point Likert	8–17	Yes^a^	1
	PROMIS v1.0 Anxiety	PROM	–	CAT	5-point Likert	≥18	Yes^a^	1
	PROMIS Pediatric v2.0 Anger 5a	PROM	–	5	5-point Likert	8–17	Yes^a^	1
	PROMIS v 2.0 Anger 5a	PROM	–	5	5-point Likert	≥18	Yes^a^	1
	PROMIS Pediatric v2.0 Depressive symptoms	PROM	–	CAT	5-point Likert	8–17	Yes^a^	1
	PROMIS v1.0 Depression	PROM	–	CAT	5-point Likert	≥18	Yes^a^	1
Relationship	PROMIS v2.0 Peer relationships	PROM	–	CAT	5-point Likert	8–17	Yes^a^	1
Social function	PROMIS v2.0 Satisfaction with social roles and activities	PROM	–	CAT	5-point Likert	≥18	Yes^a^	1
	PROMIS v2.0 Ability to participate in social roles and activities	PROM	–	CAT	5-point Likert	≥18	Yes^a^	1
HR-QOL^c^								
	PROMIS Pediatric v1.2 Global health	PROM	2	10	5-point Likert	8–17	Yes^a^	2
	PROMIS v1.2 Global health	PROM	2	10	5-point Likert	≥18	Yes^a^	2

^a^ PROMIS measures are expressed by a score relative to a reference population (T-score). The T-score is a standardized score with a mean of 50 and standard deviation of 10 in the reference population (37).

^b^ Stratified by domains according to the World Health Organization's International Classification of Functioning, disability and health (7).

^c^ Stratified by (sub)domains as described by Anthony et al. (8).

CAT, computerized adaptive testing; ClinROM, clinician-reported outcome measure; HR-QOL, health-related quality of life; PerBOM, performance-based outcome measure; PROMIS, patient-reported outcomes measurement information system; PROM, patient-reported outcome measure.

##### 2.7.2.1 Functional outcomes

The 6-Minute Walk Test (6-MWT) is employed to expand the assessment of walking. Patients walk as far as possible within a six-minute timeframe on a flat surface of at least 20 meters in length (38).

After the 6-MWT, the rate of perceived exertion (RPE) is recorded by asking the patient to rate the effort required to perform the 6-MWT on a scale from one to ten with higher numbers representing increasing intensity levels (39).

The Timed Up and Down Stairs (TUDS) is executed to further deepen the assessment of daily life activities by recording the time needed to ascend and descend one flight of stairs (40).

Functional strength of the legs is assessed by the Lateral Step Up Test (LSUT). To start this test, the patient places the leg being tested on a 15-centimeter high step, while the other leg remains on the floor. The patient is then instructed to fully extend the hip and knee of the tested leg, followed by flexion of the hip and knee until the contralateral foot touches the floor again. The final score is the number of movements within 15 seconds (41).

The ActiGraph GT9X Link (ActiGraph, LLC, Pensacola, FL) is used to objectively measure physical activity. This is a small device worn around the hip that measures step counts, acceleration (and therefore intensity of an activity) and posture. Patients are asked to wear the ActiGraph at home for seven days during day time. For adequate interpretation of results, patients are instructed to keep a brief diary noting the time periods when the ActiGraph was not worn.

The 6-MWT, RPE, TUDS and LSUT are exclusively conducted in patients in follow-up for a pelvic or lower extremity tumor. The ActiGraph is worn by all patients.

##### 2.7.2.2 Psychosocial outcomes and HR-QOL

The Patient-Reported Outcomes Measurement Information System (PROMIS) was developed by the National Institutes of Health (NIH) (42, 43). It contains a great variety of item banks for patient-reported measurements of physical, mental, and social health in children and adults. The item banks selected to evaluate problems within our bone sarcoma population are: global health, pain interference, fatigue, physical function, anxiety, anger, depressive symptoms/depression, cognitive functioning, peer relationships (children only), ability to participate in social roles and activities (adults only) and satisfaction with social roles and activities (adults only). When available, computerized adaptive testing (CAT) is used to reduce the number of items needed to complete the item bank. In the Netherlands and Belgium, the Dutch-Flemish PROMIS was created for the translation of the NIH item banks (44, 45). PROMIS is expected to dominate the field of questionnaires in the near future because of increasing use in research and expected rapid integration in patient care.

#### 2.7.3 Evaluation of multidisciplinary care

Patient satisfaction with the multidisciplinary follow-up care is assessed via a modified version of the satisfaction questionnaire developed by Blaauwbroek et al. (see Supplementary Data Sheet S4, Table 3) (46).

**Table 3:** Properties of the measurements evaluating care.

(Sub)domain(s)	Measure	Type	Subscales	Items	Response scale/units	Age (years)	Norms available	Completion time (min)
Evaluation of care								
	Satisfaction questionnaire (modified)	PROM	–	6	5-point Likert	≥8	–	2
	Actions	ClinROM	–	23	Yes/No	All	–	1

ClinROM, clinician-reported outcome measure; PROM, patient-reported outcome measure.

To record the output of care, a predefined action list is utilized, which systematically tracks the output of the clinic for each patient (see Supplementary Data Sheet S5, Table 3). Relevant action points per healthcare professional can be marked, provided that they are in response to a problem or question identified during the clinic. Actions resulting from standard information provision are not noted, since these do not indicate the clinic's added value. Examples of recorded actions include “redirection of primary care physical therapy”, “psychoeducation”, “referral to a primary care psychologist”, and “tailored advice or information provision”. Any changes in the policy of an individual healthcare professional or the establishment of a new policy based on collective consultation are also documented in the action list.

In line with the study protocol, we conducted an interim analysis of patient satisfaction after the first 60 patients were included. We expected at least 37 out of 60 patients (lower limit of the exact 95% confidence interval: 50%), meaning at least 50%, to be satisfied with the multidisciplinary clinic for it to continue. Otherwise, revisions would need to be made based on patients' feedback where necessary. Patient satisfaction was notably high, with 98% of patients reporting satisfaction with the care provided during the multidisciplinary clinic. As a result, the clinic was continued as planned.

### 2.8 Clinical data collection

Data are collected in Castor EDC, a web-based electronic data capture platform for clinical research (47). At inclusion, patients are registered in Castor via a record ID. A separate subject identification list is kept password-protected by the study coordinators. The data collected include patient characteristics such as age and sex, oncological diagnosis, treatment, and follow-up details including ClinROMs and PerBOMs completed at the clinic visit. PROMs are presented to patients via the KLIK (Quality of Life in Clinical Practice) PROM portal, a web-based platform designed for completing PROM assessments that is widely used in our hospital (9). The KLIK PROM portal enables patients and/or parents to complete assessments on HR-QOL, symptoms and psychosocial and physical functioning at home prior to scheduled outpatient consultations. The results are presented to the healthcare professionals through an ePROfile that is integrated with the patient's electronic health record. This ePROfile allows healthcare professionals to monitor and screen for potential issues and discuss the results with the patient and/or parent during consultation (48). To facilitate secure delivery of pseudonymized data from the KLIK PROM portal at study closure, the subject's record ID is entered into his/her account by the study coordinators upon enrolment.

### 2.9 Privacy

Access to the Castor database is secured by login credentials and is restricted to the principal investigator (JM), two study coordinators (LT and HiV), two supervising researchers (LH and WB), and a data manager. The data only contain pseudonymized data and are entered into the database by the study coordinators. As per hospital guidelines, data will be stored for 15 years after study closure. During the inclusion period of the study, outcomes in the KLIK PROM portal can be accessed solely by treating healthcare professionals. At study closure, a pseudonymized export file including all the data will be generated by the KLIK PROM team based on the record IDs and informed consent forms of the subjects.

### 2.10 Quality of data collection

The KLIK PROM portal requires subjects to complete all items before a PROM can be finished, preventing missing values. One of the study coordinators (LT) reviews the scoring of the adverse events following every clinic to ensure consistency and verify item completion. Inconsistencies or missing items are addressed by returning them to the healthcare professional for amendment.

### 2.11 Data analysis and general statistical considerations

Descriptive statistics will be provided for the entire group and relevant subgroups. Categorical variables will be presented as frequencies (n) and percentages (%). Continuous variables will be plotted graphically to assess distribution and determine the most appropriate method for data presentation and analysis. Means and standard deviations will be reported if variables are normally distributed. Medians and IQR's will be described otherwise. General considerations of the statistical analysis are described below. Detailed hypotheses and associated statistical analysis methods will be presented in subsequent papers stemming from this study.

The relationships between the outcomes, type of local therapy, and patient- and cancer-related factors will be explored using ANOVA, Bland-Altman analysis, or correlation coefficients, depending on the hypothesis and type of variable. If the data permits, a multiple regression analysis will be performed. Patient satisfaction and output of care will be expressed by descriptive statistics. If norm data are available, means will be compared using two sample t-tests, ANOVA or nonparametric alternatives depending on the variable. If raw norm data is available, scores will be compared by regression analysis if assumptions can be met.

Imputation will be applied to missing data for patient and tumor characteristics. The method of imputation will depend on the percentage of missing data (49). The handling of missing values for the outcome measures will be decided upon at data analysis and will be described in the subsequent papers. To account for multiple testing, the Benjamini-Hochberg procedure will be applied to mitigate the risk of false discovery.

Statistical analyses will be performed using R (50).

### 2.12 Ethical considerations

The Medical Research Ethics Committee of the University Medical Center Utrecht (Utrecht, the Netherlands) confirmed that the Medical Research Involving Human Subjects Act does not apply to this study.

In accordance with Dutch ethical regulations, informed consent must be obtained from both parents or the guardian for patients aged 15 years and younger. For patients from the age of 12, informed consent from the patient is also required. Patients aged 16 years and older are entitled to give their own consent.

## 3 Discussion

Involvement of a multidisciplinary team is essential in the care of patients with musculoskeletal sarcoma and is generally well-arranged for patients on-treatment (51). However, once patients transition to follow-up care, guidelines and logistics for maintaining this comprehensive approach are often less well established. Given the complexity and impact of local therapy and the duration and intensity of chemotherapy, it is important to continue multidisciplinary care for bone sarcoma patients after completion of primary treatment. Comprehensive and collaborative care by specialized healthcare professionals is needed to effectively identify and manage adverse events, rehabilitation challenges, and difficulties in the patients' daily life. To address this, a multidisciplinary follow-up clinic was established at our national hospital, providing a structured and systematic evaluation of bone sarcoma patients after local therapy across a wide range of outcomes. This approach enhances follow-up care for individual bone sarcoma patients, while simultaneously enabling the study of outcomes from a unique nationwide pediatric bone sarcoma cohort.

There is a growing body of literature assessing outcomes in bone sarcoma patients; however, defining clear benchmarks remains challenging due to the heterogeneity of the population and the predominance of small and/or outdated cohorts (3, 52–55). Existing studies often focus on short-term complications or chemotherapy-related events but lack a comprehensive evaluation of the permanent local therapy-related adverse events (2, 56–58). Functional outcomes are frequently assessed with limited scope, without fully covering the domains outlined by the ICF (53, 54, 59, 60). Additionally, studies comparing functional outcomes across different surgical approaches show variability in findings (61–63). This variability is also observed for psychosocial outcomes and quality of life, underscoring the need for further exploration (3).

Our study, which evaluates a large nationwide cohort of bone sarcoma patients, will significantly contribute to the existing knowledge base by providing a more comprehensive understanding of local therapy-related outcomes in follow-up. By identifying prevalent challenges, comparing surgical approaches, and exploring associated factors, we aim to enhance patient counseling, optimize intervention strategies, and take the first steps toward defining clearer benchmarks. For newly diagnosed patients, these findings will also inform the decision support models used in shared-decision making for local therapy options, and help guide postoperative care and rehabilitation to improve long-term patient outcomes.

### 3.1 Strengths and limitations

By adhering to international frameworks and incorporating the extensive expertise of our bone sarcoma experts along with literature review, this study relies on a comprehensive assessment of all the domains relevant to this patient population. Importantly, we have minimized the patient's assessment burden by aligning the study of outcomes with standardized patient care. Moreover, this systematic and comprehensive protocol creates a framework for international comparison and collaboration which is crucial for increasing our knowledge of the outcomes of bone sarcoma patients.

This study also has several limitations. First, the cross-sectional design hinders the evaluation of changes in outcomes over time. However, since the standardized assessment measures are part of our standard of care, we will collect longitudinal data in the future that could be used in a subsequent study. Second, while all childhood oncology care in the Netherlands was centralized at the Princess Máxima Center in 2018, not all patients in follow-up were transferred from the UMCs. Since this selection was based on the treating UMC rather than patient or tumor characteristics, we do not expect this to have impact on the representativeness of our cohort compared to the national pediatric bone sarcoma population. Third, despite our focus on patients with bone sarcoma of the extremities and pelvis (including 75% of bone sarcoma patients), we expect that subcohort analysis will remain limited to the most frequent bone sarcoma locations and interventions. However, by sharing our study protocol with the international community, we expect that merging internationally generated data will facilitate more detailed subcohort analysis in the near future. Finally, while this study focuses on the most prevalent bone sarcoma sites, i.e., the extremities and pelvis, we recognize the importance of multidisciplinary follow-up for patients with bone sarcomas at other locations. These patients face distinct challenges, requiring specialized assessments and healthcare professionals, making their inclusion in the current standardized clinic suboptimal. We are working toward expanding this approach to ensure comparable multidisciplinary care for all bone sarcoma patients, specifically designed for each tumor site, including head and neck and trunk.

### 3.2 Dissemination

The data will be published according to the methodology described above. After study closure, the database and subject identification list will be managed by the trial and data center of our hospital. Any future requests for the use of study data will be assessed by the trial and data center.

The study coordinators will report the results of the study as soon as possible after finalization of the data analysis through multiple publications. We will adhere to the guidelines of the publishing journal for determination of the authorships.

## 4 Conclusion

In this study, we systematically assess functional outcomes, adverse events, psychosocial outcomes, and health-related quality of life in a nationwide cohort of pediatric bone sarcoma patients in follow-up. The integrated multidisciplinary approach ensures optimal evaluation of key outcomes for these patients, while enabling transdisciplinary advice and personalized care strategies. By aligning research with standardized multidisciplinary care, we minimize the burden of additional research measurements while studying a comprehensive range of outcomes that will significantly contribute to the current knowledge base.

By sharing our study protocol, we aim to provide a framework for international collaboration generating valuable data on outcomes in this rare patient group to optimize their follow-up care. This data will also inform decision support models that enhance the shared decision-making process for patients facing life-changing local therapy, guide postoperative care and rehabilitation, and improve long-term care for future survivors.

## Data Availability

The raw data supporting the conclusions of this article will be made available by the authors, without undue reservation.
