# Supplementary material for: A multidisciplinary and structured approach for comprehensive evaluation of functional outcomes, adverse events, psychosocial outcomes and health-related quality of life after local therapy for bone sarcoma in children: protocol for a cross-sectional study
Source: Front Pediatr. 2025 Apr 15;13:1534153. doi: 10.3389/fped.2025.1534153 (PMC12037555; doi:10.3389/fped.2025.1534153)
Supplement: Supplementary file 1 [file Datasheet1.pdf]

Supplementary Data Sheet S1. Clinical report form for standardized physical assessment of upper extremities

Patient details

Princess máxima center pediatric oncology

- Patient identification:
- Age: years old
- Weight: kilograms
- Height: centimeters
- Surgery:
- Affected side: Right / Left

Functional tests

- Box & blocks test: R / L number of blocks in 60 seconds

Inspection & observation

Joint mobility

- Shoulder anteflexion (active/passive 180):	Act …… / Pass ……	R/L	Act …… / Pass ……
- Shoulder abduction (active/passive 180):	Act …… / Pass ……	R/L	Act …… / Pass ……
- Elbow flexion/extension (150-0-0):	…….. - ..…… - ..…….	R/L	…….. - ..…… - ..…….
- Elbow pronation/supination (80-0-90):	…….. - ..…… - ..…….	R/L	…….. - ..…… - ..…….
- Wrist dorsal/palmar flexion (90-0-90)	…….. - ..…… - ..…….	R/L	…….. - ..…… - ..…….
- Other joints (upon indication):			

Muscle power

- Shoulder abduction (sitting):	N		R/L	N	
- Elbow flexion (sitting):	N		R/L	N	
- Elbow extension (sitting):	N		R/L	N	
- Wrist dorsal flexion (sitting):	MRC		R/L	MRC	
- Wrist palmar flexion (sitting)	MRC		R/L	MRC	
- Pinch force:	N		R/L	N	
- Finger / hand (upon indication):					

General remarks

R / L = Right / Left; Act = Active; Pass = Passive; N = Newton; MRC = Medical Research Council scale
