# Supplementary material for: A multidisciplinary and structured approach for comprehensive evaluation of functional outcomes, adverse events, psychosocial outcomes and health-related quality of life after local therapy for bone sarcoma in children: protocol for a cross-sectional study
Source: Front Pediatr. 2025 Apr 15;13:1534153. doi: 10.3389/fped.2025.1534153 (PMC12037555; doi:10.3389/fped.2025.1534153)
Supplement: Supplementary file 2 [file Datasheet2.pdf]

Supplementary Data Sheet S2. Clinical report form for standardized physical assessment of pelvis or lower extremities

Patient details

Princess máxima center pediatric oncology

- Patient identification: [ ]
- Age: [ ] years old
- Weight: [ ] kilograms
- Height: [ ] centimeters
- Surgery: [ ]
- Affected side: Right / Left

Inspection & observation

Leg length discrepancy: No / Yes [ ] centimeters

Joint mobility (range)

- Hip flexion / extension (145-0-10):	…….. - …….. - ……..	R/L	…….. - …….. - ……..
- Knee flexion / extension (145-0-0):	…….. - …….. - ……..	R/L	…….. - …….. - ……..
- Ankle dorsal / plantar flexion (20-0-50):	…….. - …….. - ……..	R/L	…….. - …….. - ……..
- Patellar mobility (in extension):	-- / - / = / + / ++	R/L	-- / - / = / + / ++
- Patella alta (in 90 degrees flexion):	-- / - / = / + / ++	R/L	-- / - / = / + / ++
- Other joints (upon indication):			

Muscle length (angle)

- Calf muscle length (straightened knee):	°	R/L	°
- Hamstrings length (popliteal angle):	°	R/L	°

Muscle power

- Extensor lag (sitting):		°	R/L		°
- Hip extension (prone position):	MRC		R/L	MRC	
- Hip abduction (lateral position):	MRC		R/L	MRC	
- Knee flexion:	N		R/L	N	
- Knee extension:	N		R/L	N	
- Dorsal flexion ankle:	MRC		R/L	MRC	
- Plantar flexion ankle:	MRC		R/L	MRC	
- Functioning of additional muscles (upon indication):					
- Remarks:					

Functional tests

- Toe walking: [ ]
- Heel walking: [ ]
- Stand on one leg: [ ] R / L [ ] duration in seconds (max 120 sec)
- 10 meter walk test: [ ] seconds
- 10 meter run test: [ ] seconds
- *Lateral step up test*:* [ ] *R / L* [ ] *number in 15 seconds*
- *Timed-up-down-stairs*:* [ ] *duration in seconds*
- *6-minute walk test*:* [ ] *meters*
- *Rate of perceived exertion*:* [ ] *score*

General remarks

Only performed when the patient has consented for additional research assessments

R / L = Right / Left; N = Newton; MRC = Medical Research Council scale
