# Supplementary material for: A multidisciplinary and structured approach for comprehensive evaluation of functional outcomes, adverse events, psychosocial outcomes and health-related quality of life after local therapy for bone sarcoma in children: protocol for a cross-sectional study
Source: Front Pediatr. 2025 Apr 15;13:1534153. doi: 10.3389/fped.2025.1534153 (PMC12037555; doi:10.3389/fped.2025.1534153)
Supplement: Supplementary file 3 [file Datasheet3.pdf]

Supplementary Data Sheet S3. Selection of CTCAE items stratified by tumor location and type of local therapy

**CTCAE items**	**Upper extremity Surgery only**	**Upper extremity RTx ± surgery**	**Pelvis Surgery only**	**Pelvis RTx ± surgery**	**Lower extremity Surgery only**	**Lower extremity RTx ± surgery**
Gastrointestinal disorders						
Colitis	-	-	-	x	-	-
Constipation	-	-	-	x	-	-
Diarrhoea	-	-	-	x	-	-
Enterocolitis	-	-	-	x	-	-
Faecal incontinence	-	-	-	x	-	-
Fistula	-	-	-	x	-	-
Malabsorption	-	-	-	x	-	-
Proctitis	-	-	-	x	-	-
Small intestinal perforation	-	-	-	x	-	-
Gastrointestinal disorders - Other, specify	-	-	-	x	-	-
General disorders and administration site conditions						
Gait disturbance	-	-	x	x	x	x
Pain (including neurogenic and phantom pain)	x	x	x	x	x	x
Infections and infestations						
Infections	x	x	x	x	x	x
Injury, poisoning and procedural complications						
Fracture	x	x	x	x	x	x
Musculoskeletal and connective tissue disorders						
Arthritis	x	x	x	x	x	x
Avascular necrosis	x	x	x	x	x	x
Joint effusion	x	x	x	x	x	x
Joint range of motion decreased	x	x	x	x	x	x
Kyphosis	-	-	x	x	-	-
Lordosis	-	-	x	x	-	-
Muscle weakness lower limb	-	-	x	x	x	x
Muscle weakness trunk	x	x	x	x	-	-
Muscle weakness upper limb	x	x	-	-	-	-
Musculoskeletal deformity	x	x	x	x	x	x
Osteonecrosis	x	x	x	x	x	x

Rotator cuff injury	x	x	-	-	-	-
Scoliosis	-	-	x	x	-	-
Unequal limb length	x	x	x	x	x	x
Musculoskeletal and connective tissue disorder - Other, specify	x	x	x	x	x	x
Neoplasms benign, malignant and unspecified						
Treatment related secondary malignancy	-	x	-	x	-	x
Nervous system disorders						
Brachial plexopathy	x	x	-	-	-	-
Dysesthesia	x	x	x	x	x	x
Paraesthesia	x	x	x	x	x	x
Peripheral motor neuropathy	x	x	x	x	x	x
Peripheral sensory neuropathy	x	x	x	x	x	x
Nervous system disorders - Other, specify	x	x	x	x	x	x
Pregnancy, puerperium and perinatal conditions						
Pregnancy, puerperium and perinatal conditions - Other, specify	-	-	-	x	-	-
Renal and urinary disorders						
Cystitis non-infective	-	-	-	x	-	-
Renal and urinary disorders - Other, specify	-	-	-	x	-	-
Reproductive system and breast disorders						
Amenorrhea	-	-	-	x	-	-
Dyspareunia	-	-	-	x	-	-
Irregular menstruation	-	-	-	x	-	-
Pelvic floor muscle weakness	-	-	-	x	-	-
Premature menopause	-	-	-	x	-	-
Vaginal dryness	-	-	-	x	-	-
Reproductive system and breast disorders - Other, specify	-	-	-	x	-	-
Skin and subcutaneous tissue disorders						
Alopecia	x	x	x	x	x	x
Dry skin	x	x	x	x	x	x
Fat atrophy	x	x	x	x	x	x

Skin atrophy	x	x	x	x	x	x
Skin hyperpigmentation	x	x	x	x	x	x
Skin hypopigmentation	x	x	x	x	x	x
Skin induration	x	x	x	x	x	x
Telangiectasia	x	x	x	x	x	x
Skin and subcutaneous tissue disorders - Other, specify	x	x	x	x	x	x
Vascular disorders						
Lymphedema	x	x	x	x	x	x
Vascular disorders - Other, specify	x	x	x	x	x	x
