# Supplementary material for: A multidisciplinary and structured approach for comprehensive evaluation of functional outcomes, adverse events, psychosocial outcomes and health-related quality of life after local therapy for bone sarcoma in children: protocol for a cross-sectional study
Source: Front Pediatr. 2025 Apr 15;13:1534153. doi: 10.3389/fped.2025.1534153 (PMC12037555; doi:10.3389/fped.2025.1534153)
Supplement: Supplementary file 4 [file Datasheet4.pdf]

Supplementary Data Sheet S4. Satisfaction questionnaire modified from Blaauwbroek et al.

	**Very satisfied**	**Satisfied**	**Neutral**	**Not satisfied**
Satisfied with care given by healthcare professionals	☐	☐	☐	☐
Satisfied with time available during screening by healthcare professionals	☐	☐	☐	☐
Satisfied with healthcare professionals' knowledge of my medical history	☐	☐	☐	☐
Healthcare professionals' attitude was friendly	☐	☐	☐	☐
Satisfied with answers given by healthcare professionals	☐	☐	☐	☐
Able to ask all questions regarding health status	☐	☐	☐	☐
