# Supplementary material for: A multidisciplinary and structured approach for comprehensive evaluation of functional outcomes, adverse events, psychosocial outcomes and health-related quality of life after local therapy for bone sarcoma in children: protocol for a cross-sectional study
Source: Front Pediatr. 2025 Apr 15;13:1534153. doi: 10.3389/fped.2025.1534153 (PMC12037555; doi:10.3389/fped.2025.1534153)
Supplement: Supplementary file 5 [file Datasheet5.pdf]

**Supplementary Data Sheet S5.** Action list

| | Action points | |
|---|---|---|
| *Paediatric oncology or Late effects* | Referral to primary care, namely… | ☐ |
| | Referral secondary/tertiary care, namely… | ☐ |
| | Supplementary diagnostics, namely… | ☐ |
| | Other, namely… | ☐ |
| *Orthopaedics* | Information provision / advice | ☐ |
| | Indication for surgical revision of current reconstruction | ☐ |
| | Indication for correction of limb length discrepancy | ☐ |
| | Supplementary diagnostics, namely… | ☐ |
| | Other, namely… | ☐ |
| *Rehabilitation & Physical therapy* | Information provision/advice | ☐ |
| | Redirection of primary care (e.g. first-line physical therapy) | ☐ |
| | Referral to primary care, namely… | ☐ |
| | Referral to secondary care / rehabilitation centre, namely… | ☐ |
| | Supplementary diagnostics, namely… | ☐ |
| | Other, namely… | ☐ |
| *Psychology* | Psycho-education | ☐ |
| | Follow-up appointment | ☐ |
| | Referral primary care psychologist | ☐ |
| | Referral secondary care psychologist | ☐ |
| | Supplementary diagnostics, namely… | ☐ |
| | Other, namely… | ☐ |
| *Multidisciplinary team* | New action point defined, namely… | ☐ |
| | Change in an individual healthcare professional's policy after team meeting, namely… | ☐ |
